# Risk of Obstructive Sleep Apnea in Adult Patients with Asthma: A Population-Based Cohort Study in Taiwan

**DOI:** 10.1371/journal.pone.0128461

**Published:** 2015-06-11

**Authors:** Te-Chun Shen, Cheng-Li Lin, Chang-Ching Wei, Chia-Hung Chen, Chih-Yen Tu, Te-Chun Hsia, Chuen-Ming Shih, Wu-Huei Hsu, Fung-Chang Sung, Chia-Hung Kao

**Affiliations:** 1 Graduate Institute of Clinical Medicine Science, College of Medicine, China Medical University, Taichung, Taiwan; 2 Division of Pulmonary and Critical Care Medicine, Department of Internal Medicine, China Medical University Hospital, Taichung, Taiwan; 3 Management Office for Health Data, China Medical University Hospital, Taichung, Taiwan; 4 Division of Nephrology, Department of Pediatrics, China Medical University Hospital, Taichung, Taiwan; 5 Department of Nuclear Medicine and PET Center, China Medical University Hospital, Taichung, Taiwan; University of Athens, GREECE

## Abstract

**Background:**

There are several publications reported that obstructive sleep apnea (OSA) was associated with asthma. However, large-scaled, population-based cohort study has been limited. We aimed to examine the risk of OSA among adult patients with asthma in an Asian population.

**Methods:**

We conducted a retrospective cohort study using data from the National Health Insurance (NHI) of Taiwan. The asthma cohort included 38,840 newly diagnosed patients between 2000 and 2010. The date of diagnosis was defined as the index date. Each patient was randomly matched with four people without asthma according to gender, age, and the index year as the comparison cohort. The occurrence of OSA was followed up until the end of 2011. The risk of OSA was estimated using the Cox proportional hazard model after adjusting for gender, age, and comorbidities.

**Results:**

The overall incidence of OSA was 2.51-fold greater in the asthma cohort than in the comparison cohort (12.1 versus 4.84 per 1000 person-years). Compared to non-asthma subjects, the adjusted hazard ratio (aHR) of OSA increased to 1.78 for asthma patients with one or less annual emergency room (ER) visit, and 23.8 for those who visited ER more than once per year. In addition, aHR in patients with inhaled steroid treatment compared to those without steroid treatment was 1.33 (95% CI = 1.01–1.76).

**Conclusion:**

Patients with asthma have a significantly higher risk of developing OSA than the general population. The results suggest that the risk of OSA is proportional to asthma control and patients with inhaled steroid treatment have a higher risk for OSA than those without steroid treatment.

## Introduction

Asthma is a serious global health problem affecting all age groups. It is a heterogeneous disease, usually characterized by chronic airway inflammation. The definition and diagnosis are based on the history of characteristic symptoms and evidence of variable airflow limitation [1]. Certain comorbidities are commonly present in patients with asthma, particularly those with difficult-to-treat asthma. The most common comorbid diseases include gastroesophageal reflux disease (GERD), rhinitis, sinusitis, anxiety, and depression [2–5]. In addition, patients with asthma often have poor sleep quality, which contributes to nocturnal deterioration of typical symptoms or other specific features [6].

Obstructive sleep apnea (OSA) is characterized by repeated episodes of upper airway obstruction that results in brief periods of breathing cessation or a marked reduction in airflow during sleep [7]. OSA is a common disorder with an estimated prevalence of 10%–20% [8]. Risk factors for OSA include male gender, age, obesity, and nasal diseases such as rhinitis [9]. Patients with OSA tend to have circular upper airways, whereas normal people have elliptical upper airways [10]. The most common type of upper airway obstruction is velopharyngeal narrowing, which accounts for about 80% of all cases [11]. Diagnosis of OSA is essential because of the subsequent cardiovascular comorbidities and the risk of sudden death [12].

Several publications have discussed relationships between asthma and OSA [13–17]. Salles et al. reported that OSA is prevalent in patients with asthma and is associated with disease severity. They considered OSA to be one of the most important pathophysiological mechanisms related to the worsening of asthma symptoms [13]. Treatment of OSA has been shown to improve asthma symptoms [18–19]. The National Asthma Education and Prevention Program's Expert Panel Report 3 have recommend OSA evaluation in patients with asthma because OSA is a potential factor for asthma control [20]. However, large-scaled, population-based cohort study has been limited.

Taiwan’s National Health Insurance (NHI) database is a nationwide cohort dataset, which provides reliable data and has been used for various studies on either asthma or OSA [21–24]. In the present study, we aimed to determine whether asthma is associated with an increased risk of OSA.

## Materials and Methods

### Data source

The NHI program has been active in Taiwan since March 1, 1995. According to the NHI annual statistics report, the coverage rate of NHI in 2007 was nearly 99% of the entire population of Taiwan, and more than 25 million people were enrolled in this program (http://www.nhi.gov.tw/english/index.aspx). This population-based cohort study was conducted using registration and claims datasets from 2000 to 2011 obtained from the Longitudinal Health Insurance Database 2000 (LHID2000), a subset of the National Health Insurance Research Database (NHIRD), which is managed by Taiwan's National Health Research Institutes (NHRI). The LHID2000 contains all ambulatory and inpatient claims data of one million beneficiaries who were randomly sampled from the 2000 registry for beneficiaries of the NHIRD. All personal information was encoded to protect privacy with surrogate identification before release for this research. Diagnostic codes were based on the International Classification of Diseases, ninth revision, Clinical Modification (ICD-9-CM). This study was exempted from full ethical review by the International Review Board, China Medical University and Hospital Research Ethics Committee (IRB permit number: CMU-REC-101-012).

### Study participants

Using the LHID2000 from 2000–2010, we enrolled patients above 20 years, who had been diagnosed with asthma (ICD-9 code 493), as the asthma cohort. Exclusion criteria included those diagnosed with OSA (ICD-9 code 327.23, 780.51, 780.53, 780.57) [23, 24] before index date, and with incomplete gender or age information. The index date was defined as the date of asthma diagnosis. The comparison cohort was randomly selected from all NHI beneficiaries, non-asthma, above 20 years, and was frequency-matched for gender, age (every five years), and index year with a 1:4 ratio. The diagnosis of asthma was made based on a target history, and a comprehensive pulmonary function evaluation. Physicians usually follow the GINA guideline [1] for diagnosis. Similarly, the diagnosis of OSA always needs standard polysomnography [the common diagnostic criteria was apnea-hypopnea index (AHI) >5].

### Outcome and relevant variables

All subjects were followed up until the occurrence of OSA; withdrawal from NHI; death; or December 31 2011; whichever came first. The OSA related co-morbidities included hypertension (ICD-9 code 401–405), diabetes (ICD-9 code 250), hyperlipidemia (ICD-9 code 272), chronic obstructive pulmonary disease (COPD) (ICD-9 code 496), coronary artery disease (CAD) (ICD-9 code 410–414), stroke (ICD-9 code 430–438), rhinitis (ICD-9 code 472–477), chronic sinusitis (ICD-9-CM 473), GERD (ICD-9 code 530.11, 530.81), and obesity (ICD-9 code 278). All diseases were confirmed only while those diagnostic codes that appeared at least twice within a year.

### Statistical analysis

The differences in demographic characteristics, and comorbidities between the asthma, and comparison cohorts were examined using Chi-square test for categorical variables, and Student’s *t*-test for continuous variables. Incidence density rates were calculated by dividing the number of patients with OSA by total person years of follow-up. The asthma-to-comparison hazard ratios (HRs) and 95% confidence interval (CIs) for OSA by age-, sex-, and comorbidity-specific were calculated using univariable and multivariable Cox proportion hazard regression models. Only confounding variables that were found to be significant in the multivariable model were further analyzed. The combined effects of comorbidity and asthma were also assessed in the Cox models. We further analyzed the effect of the status of asthma control on the risk of OSA, based on the number of emergency room (ER) visits for asthma. In addition, we performed Cox proportional hazards regression analysis to measure hazard ratio of OSA among asthma patients by different treatments. We plotted the Kaplan-Meier curve to estimate the cumulative incidence of subsequent OSA between the asthma and comparison cohorts. Log-rank test was used to examine the significance of difference between the two cohorts. All statistical analyses were performed using SAS 9.3 statistical software (SAS Institute, Inc., Cary, NC, USA). A p value < 0.05 was considered to be significant.

## Results

**Fig 1:**
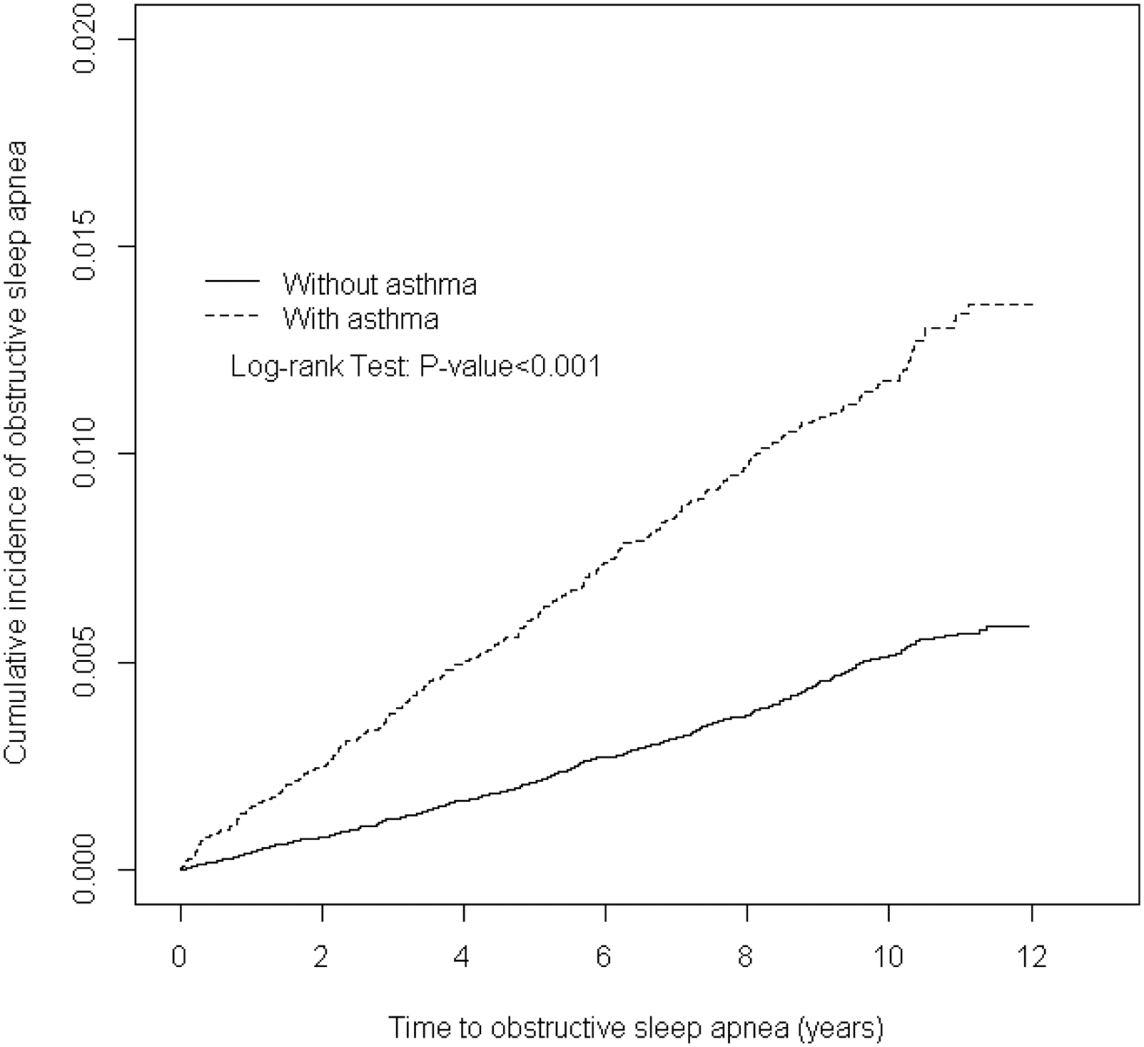
Comparison of cumulative incidence of obstructive sleep apnea between patients with or without asthma.

We enrolled 38,840 patients with asthma from 2000–2010 as the asthma cohort, and 155,347 gender- and age-matched patients without asthma as the comparison cohort. Table 1 displays the difference in demographic characteristics, and comorbidities among the two cohorts. The mean age of the asthma cohort was 52.8 years (SD = 18.1), and the comparison cohort was 53.3 years (SD = 18.0). The asthma cohort had a significantly higher rate of hypertension, diabetes, hyperlipidemia, COPD, CAD, stroke, rhinitis, chronic sinusitis, GERD and obesity (all p < 0.001) than the comparison cohort. The mean follow-up period was 6.95 years (SD = 3.33) for the asthma cohort, and 6.51 years (SD = 3.44) for the comparison cohort. Fig 1 displays the results of Kaplan-Meier survival analysis between patients with or without asthma. The log-rank test showed that the asthma cohort had significantly higher cumulative incidence rates of OSA than the comparison cohort (p < 0.001, Fig 1).

**Table 1:** Comparisons in demographic characteristics and comorbidities between cohorts with and without asthma.

Variables	Asthma				
	No		Yes		
	(N = 155347)		(N = 38840)		
	n	%	n	%	p-value^†^
**Sex**					0.99
Female	84776	54.6	21194	54.6	
Male	70571	45.4	17646	45.4	
**Age, years**					0.99
20–34	30004	19.3	7501	19.3	
35–49	36868	23.7	9217	23.7	
50–64	41204	26.5	10301	26.5	
≥ 65	47271	30.4	11821	30.4	
Mean (SD)^†^	52.8 (18.1)		53.3 (18.0)		<0.001
**Comorbidity**					
Hypertension	46377	29.9	15898	40.9	<0.001
Diabetes	12982	8.30	3672	9.45	<0.001
Hyperlipidemia	25921	16.7	8781	22.6	<0.001
COPD	2653	1.71	3786	9.75	<0.001
CAD	30901	19.9	11828	30.5	<0.001
Stroke	5740	3.69	2071	5.33	<0.001
Rhinitis	16262	10.5	13525	34.8	<0.001
Chronic sinusitis	2171	1.40	1507	3.88	<0.001
GERD	1360	0.88	752	1.94	<0.001
Obesity	1269	0.82	675	1.74	<0.001

COPD, chronic obstructive pulmonary disease; CAD, coronary artery disease; GERD, gastroesophageal reflux disease; Chi-square test;

^†^Two sample t-test

The overall incidence of OSA was 2.51–fold higher in the asthma cohort than in the comparison cohort (12.1 vs. 4.84 per 1,000 person-years) (Table 2). After adjusting for gender, age, and comorbidities, the HR for developing OSA during the follow-up years was 1.87 (95% CI = 1.61–2.17) for the asthma cohort as compared to the comparison cohort. The incidence of OSA was higher in men than in women in both cohorts. The adjusted HRs of OSA were 2.14 (95% CI = 1.77–2.59) and 1.50 (95% CI = 1.17–1.92) in men and women, respectively. The age-specific adjusted HRs of OSA for asthma cohort to comparison cohort were significant for all age subgroups. The incidence of OSA was higher in those with any comorbidity than in those without comorbidity in both cohorts. The adjusted HRs of OSA were 2.05 (95% CI = 1.74–2.41) and 1.90 (95% CI = 1.40–2.59) in those with and without comorbidity, respectively.

**Table 2:** Incidence of obstructive sleep apnea and Cox method estimated hazard ratio of obstructive sleep apnea for asthma cohort compared with non-asthma cohort by demographic characteristics and comorbidity.

Variables	Asthma							
	No (N = 155347)			Yes (N = 38840)			Crude HR^#^ (95%CI)	Adjusted HR^†^ (95%CI)
	Event	Person years	rate^$^	Event	Person years	rate^$^		
Total	521	1076209	4.84	328	269876	12.1	2.51 (2.19, 2.89)***	1.87 (1.61, 2.17)***
**Sex**								
Female	229	596672	3.84	111	150340	7.38	1.93 (1.54, 2.42)***	1.50 (1.17, 1.92)**
Male	292	479537	6.09	217	119537	18.2	2.98 (2.50, 3.56)***	2.14 (1.77, 2.59)***
**Age, years**								
20–34	72	212911	3.38	45	54752	8.22	2.43 (1.67, 3.52)***	1.63 (1.07, 2.49)*
35–49	136	271160	5.02	103	68497	15.0	3.00 (2.32, 3.88)***	1.91 (1.44, 2.54)***
50–64	174	297741	5.84	120	74626	16.1	2.76 (2.18, 3.48)***	2.00 (1.55, 2.58)***
≥ 65	139	294397	4.72	60	72001	8.33	1.77 (1.31, 2.39)***	1.42 (1.03, 1.97)*
**Comorbidity** ^**‡**^								
No	209	604795	3.46	50	75489	6.62	1.90 (1.40, 2.59)***	1.90 (1.40, 2.59)***
Yes	312	471414	6.62	278	194387	14.3	2.16 (1.84, 2.54)***	2.05 (1.74, 2.41)***

Rate^$^ per 1000 person-year; Crude HR^#^, relative hazard ratio;

^†^ Model was adjusted for age, sex, and comorbidities of hypertension, diabetes, hyperlipidemia, COPD, CAD, stroke, rhinitis, chronic sinusitis, GERD and obesity;

^‡^ Patients with any comorbidity of hypertension, diabetes, hyperlipidemia, COPD, CAD, stroke, rhinitis, chronic sinusitis, GERD, and obesity was defined as the comorbidity group;

*p<0.05,

**p<0.01,

***p<0.001

Table 3 shows the combined effects of asthma and comorbidities on the risk of OSA. When compared to subjects without asthma and GERD, the adjusted HR increased to 11.4 (95% CI = 6.68–19.4) for subjects with asthma and GERD. Co-existence with obesity (adjusted HR: 6.07, 95% CI = 3.24–11.4), hyperlipidemia (adjusted HR: 4.80, 95% CI = 3.88–5.94), rhinitis (adjusted HR: 4.29, 95% CI = 3.57–5.14), hypertension (adjusted HR: 4.11, 95% CI = 3.33–5.07) and CAD (adjusted HR: 4.10, 95% CI = 3.31–5.08) enhanced the risk of OSA in patients with asthma.

**Table 3:** Cox proportional hazard regression analysis for the risk of obstructive sleep apnea-associated asthma with combined effect of comorbidity.

Variables		Event		Adjusted HR^†^	
		N	n	(95% CI)	p-value^#^
Asthma	Hypertension				0.13
No	No	108970	315	1 (Reference)	
No	Yes	46377	206	1.93 (1.58, 2.36)***	
Yes	No	22942	174	2.56 (2.13, 3.09)***	
Yes	Yes	15898	154	4.11 (3.33, 5.07)***	
Asthma	Hyperlipidemia				0.45
No	No	129426	395	1 (Reference)	
No	Yes	2592	126	1.83 (1.49, 2.25)***	
Yes	No	30059	213	2.32 (1.96, 2.74)***	
Yes	Yes	8781	115	4.80 (3.88, 5.94)***	
Asthma	CAD				0.14
No	No	124446	371	1 (Reference)	
No	Yes	30901	150	2.02 (1.65, 2.47)***	
Yes	No	27012	207	2.53 (2.13, 3.00)***	
Yes	Yes	11828	121	4.10 (3.31, 5.08)***	
Asthma	Rhinitis				0.96
No	No	139085	437	1 (Reference)	
No	Yes	16262	84	2.07 (1.64, 2.62)***	
Yes	No	25315	166	2.03 (1.70, 2.43)***	
Yes	Yes	13525	162	4.29 (3.57, 5.14)***	
Asthma	GERD				0.06
No	No	153987	518	1 (Reference)	
No	Yes	1360	3	1.42 (0.46, 4.42)	
Yes	No	38088	314	2.44 (2.12, 2.80)***	
Yes	Yes	752	14	11.4 (6.68, 19.4)***	
Asthma	Obesity				0.49
No	No	154078	511	1 (Reference)	
No	Yes	1269	10	3.25 (1.74, 6.07)***	
Yes	No	38165	318	2.51 (2.18, 2.88)***	
Yes	Yes	675	10	6.07 (3.24, 11.4)***	

COPD, chronic obstructive pulmonary disease; CAD, coronary artery disease; GERD, gastroesophageal reflux disease;

^†^ Model was adjusted for age and sex;

^#^ p-value for interaction;

***p<0.001

Table 4 shows that the adjusted HR of OSA increased to 1.78 (95% CI = 1.53–2.08) for patients with asthma with one or less ER visit per year, and 23.8 (95% CI = 14.5–39.0) for those who visited ER more than one times per year (*p* for trend < 0.0001). Table 5 shows that incidence of OSA was highest in asthma patients with inhaled steroid treatment (15.3 per 1000 person-years), and adjusted HR was 1.33 (95% CI = 1.01–1.76) in patients with inhaled steroid treatment compared to those without steroid treatment.

**Table 4:** Hazard ratios of obstructive sleep apnea associated with the number of annual emergency room visits for patients with asthma.

	No. of Event	Crude HR^#^ (95% CI)	Adjusted HR^†^ (95% CI)
Non-asthma	521	1 (Reference)	1 (Reference)
Times of annual emergency room visit			
≤1	311	2.39 (2.08, 2.76)***	1.78 (1.53, 2.08)***
>1	17	30.9 (19.0, 50.2)***	23.8 (14.5, 39.0)***
*p* for trend		<0.001	<0.001

Crude HR^#^, relative hazard ratio;

^†^ Model was adjusted for age, sex, and comorbidities of hypertension, diabetes, hyperlipidemia, COPD, CAD, stroke, rhinitis, chronic sinusitis, GERD and obesity;

***p<0.001

**Table 5:** Cox proportional hazards regression analysis measured hazard ratio of obstructive sleep apnea among asthma patients by different treatments.

Variables	N	Event	PY	Rate^$^	Crude HR^#^ (95% CI)	Adjusted HR^†^ (95% CI)
Treatments of asthma						
Non-steroid	13792	89	84271	10.6	1 (Reference)	1 (Reference)
Inhaled steroid	11214	128	83602	15.3	1.46 (1.11, 1.92)**	1.33 (1.01, 1.76)*
Systemic steroid	13834	111	102003	10.9	1.04 (0.79, 1.37)	1.01 (0.76, 1.34)

Rate^$^ per 1000 person-year; Crude HR^#^, relative hazard ratio;

^†^ Model was adjusted for age, sex, and comorbidities of hypertension, diabetes, hyperlipidemia, COPD, CAD, stroke, rhinitis, chronic sinusitis, GERD and obesity;

*p<0.05,

**p<0.01

## Discussion

This population-based cohort study demonstrates an increased risk of OSA in patients with asthma as compared to the general population (12.1 versus 4.84 per 1000 person-years; crude HR: 2.51; 95% CI = 2.19–2.89; p < 0.001). This finding is compatible with the well-known concept that male participants, older subjects, and those with risk factor such as cardiovascular diseases, stroke, COPD, rhinitis, sinusitis, GERD, and obesity may have a higher incidence of OSA. In the present study, we found adjusted HRs were greater in men than in women (2.14 vs. 1.54), in 50–64 years age group than in other age groups (2.00 vs. 1.63, 1.19 and 1.42), and in those with any comorbidities than in those without comorbidity (2.05 vs. 1.90). In addition, our study showed that the risk of developing OSA increased proportionately with the number of annual ER visits for asthma (adjusted HR from 1.78 to 23.8).

Several cross-sectional population-based studies have described the prevalence of OSA based on questionnaires submitted by people with asthma. Fitzpatrick MF et al. reported that people with asthma of all ages and body weights snored more often than the controls in a British population [25]. In the European Community Health Respiratory Survey (Iceland, Sweden, and Belgium), Janson et al. noticed that self-reported snoring and apnea were significantly more common in young adults with asthma versus controls (14% vs 9% and 3% vs 1%, respectively) [26]. Larsson LG et al. in a Swedish study found 17% snoring and 14% apnea in those with asthma, as compared to an overall prevalence of 10% and 7%, respectively [27]. Similarly, Ekici et al. conducted a study in Turkey involving 7469 adults; of which, 2713 had a history of asthma. They reported that snoring (OR = 1.7) and apnea (OR = 2.7) were more prevalent in patients with asthma than those without asthma [28]. However, these studies on association between asthma and OSA were cross-sectional studies, and the findings may not be a valid reflection of the true risk of OSA associated with asthma. In the present study, we identified that the overall incidence of OSA was 2.51-fold higher in the asthma cohort than in the comparison cohort, and a multivariable Cox method measured an adjusted HR of 1.87.

Several hypotheses have been postulated on the interactions between asthma and OSA. First, shared risks and comorbid conditions, such as GERD, rhinitis, and obesity, could play a very important role [13–16]. For example, Braido F et al. reported that 52.6% (1021/1941) of asthma subjects had OSA, including 47.3% (350/740) with asthma alone and 55.9% (671/1201) with asthma and allergic rhinitis [16]. In the present study, we also noticed strong combined effects of these comorbidities and asthma on the risk of OSA, particularly those with GERD (adjusted HR: 11.4) and with obesity (adjusted HR: 6.07). Second, systemic inflammation may contribute to both asthma and OSA. Asthma is associated with acute and chronic inflammation, which affects the strength or force generation of the respiratory muscles, including the upper airway dilator muscles [29]. The biological mechanism linking lower airway inflammation with sleep-related upper airway collapse may explain a unified airway hypothesis. Other theories include sleep architecture (neural), tracheal tug (mechanical), and respiratory phase interdependence (mechanical) [14]. In addition, corticosteroid usage for asthma control may also influence the incidence of OSA [30, 31]. In the present study, our findings also support the notion that asthma patients with inhaled steroid treatment have a higher risk for OSA development than patients without steroid treatment.

Some studies focused on the relationships between moderate to severe or difficult-to-treat asthma and OSA [7, 32–33]. Most of them suggested that OSA contributes to worsen asthma control. Recently, Teodorescu M et al. reported asthma was associated with an increased risk of new-onset OSA and they found asthma-OSA association was significantly dose-dependent on duration of asthma [17]. However, the correlation between asthma control and OSA incidence remains unknown. In the present study, the number of ER visits per year for asthma has been considered to reflect, at least in part, the asthma control status. The results related to ER visits also supported the hypothesis that poor control of asthma results in an increased risk of OSA. In addition, subjects with inhaled steroid control may also indicate a more difficult-to-control group of asthma (at least step 2 in GINA guideline). They do indeed have a higher risk for OSA development than patients without steroid treatment.

The strength of this study is in providing a longitudinal population-based evaluation of Asians with asthma, and their risks of developing OSA. It is generally very costly to conduct a population-based prospective cohort study, in which loss to follow-up is problematic after several years. Therefore, a retrospective cohort study using insurance or register data is an alternative, which meets the requirement but is economical. However, there are several limitations to be considered when interpreting the present findings. First, this study used the ICD-9-CM algorithm to define asthma, OSA, and comorbidities. The diagnosis depends on the performance of clinical physicians. An ad hoc committee established by the insurance authority was in charge of evaluating the claims data to prevent errors and violation. In addition, we selected only those diagnoses that appeared at least twice within a year to increase the validity and accuracy of diagnosis. Second, NHRID does not provide detailed information on severity of asthma, socioeconomic status, environmental factors, occupation, smoking habits, alcohol consumption, abdominal adiposity indices, such as waist circumference (WC) or waist-to-height ratio, body mass index, diet preference, physical activity, and family history, although these are potential confounding factors for this study. In addition, relevant clinical variables, such as serum laboratory data, polysomnography, pulmonary function tests, or imaging results of patients were unavailable in our study.

## Conclusion

Patients with asthma have a significantly higher risk of developing OSA than the general population. The results suggest that the risk of OSA is proportional to asthma control and patients with inhaled steroid treatment have a higher risk for OSA than patients without steroid treatment. Since evaluating OSA in patients with asthma has been included in the Guidelines for the Diagnosis and Management of Asthma [20], we suggest that periodic OSA evaluation in certain asthma patients may have a clinical benefit in the management of both asthma and OSA.
