# Correction: Risk of Obstructive Sleep Apnea in Adult Patients with Asthma: A Population-Based Cohort Study in Taiwan

**DOI:** 10.1371/journal.pone.0219612

**Published:** 2019-07-05

**Authors:** Te-Chun Shen, Cheng-Li Lin, Chang-Ching Wei, Chia-Hung Chen, Chih-Yen Tu, Te-Chun Hsia, Chuen-Ming Shih, Wu-Huei Hsu, Fung-Chang Sung, Chia-Hung Kao

There is an error in the Table 2 footnotes: “Rate^$^ per 1000 person-year” should appear as “Rate^$^ per 10000 person-year.”

**Table 2:** Incidence of obstructive sleep apnea and Cox method estimated hazard ratio of obstructive sleep apnea for asthma cohort compared with non-asthma cohort by demographic characteristics and comorbidity.

Variables	Asthma							
	No (N = 155347)			Yes (N = 38840)			Crude HR^#^ (95%CI)	Adjusted HR^†^ (95%CI)
	Event	Person years	rate^$^	Event	Person years	rate^$^		
Total	521	1076209	4.84	328	269876	12.1	2.51 (2.19, 2.89)***	1.87 (1.61, 2.17)***
**Sex**								
Female	229	596672	3.84	111	150340	7.38	1.93 (1.54, 2.42)***	1.50 (1.17, 1.92)**
Male	292	479537	6.09	217	119537	18.2	2.98 (2.50, 3.56)***	2.14 (1.77, 2.59)***
**Age, years**								
20–34	72	212911	3.38	45	54752	8.22	2.43 (1.67, 3.52)***	1.63 (1.07, 2.49)*
35–49	136	271160	5.02	103	68497	15.0	3.00 (2.32, 3.88)***	1.91 (1.44, 2.54)***
50–64	174	297741	5.84	120	74626	16.1	2.76 (2.18, 3.48)***	2.00 (1.55, 2.58)***
≥ 65	139	294397	4.72	60	72001	8.33	1.77 (1.31, 2.39)***	1.42 (1.03, 1.97)*
**Comorbidity**^**‡**^								
No	209	604795	3.46	50	75489	6.62	1.90 (1.40, 2.59)***	1.90 (1.40, 2.59)***
Yes	312	471414	6.62	278	194387	14.3	2.16 (1.84, 2.54)***	2.05 (1.74, 2.41)***

Rate^$^ per 10000 person-year; Crude HR#, relative hazard ratio;

† Model was adjusted for age, sex, and comorbidities of hypertension, diabetes, hyperlipidemia, COPD, CAD, stroke, rhinitis, chronic sinusitis, GERD and obesity;

‡ Patients with any comorbidity of hypertension, diabetes, hyperlipidemia, COPD, CAD, stroke, rhinitis, chronic sinusitis, GERD, and obesity was defined as the comorbidity group;

*p<0.05,

**p<0.01,

***p<0.001

There is an error in the Table 5 footnotes: “Rate^$^ per 1000 person-year” should appear as “Rate^$^ per 10000 person-year.”

**Table 5:** Cox proportional hazards regression analysis measured hazard ratio of obstructive sleep apnea among asthma patients by different treatments.

Variables	N	Event	PY	Rate^$^	Crude HR^#^ (95% CI)	Adjusted HR^†^ (95% CI)
Treatments of asthma						
Non-steroid	13792	89	84271	10.6	1 (Reference)	1 (Reference)
Inhaled steroid	11214	128	83602	15.3	1.46 (1.11, 1.92)**	1.33 (1.01, 1.76)*
Systemic steroid	13834	111	102003	10.9	1.04 (0.79, 1.37)	1.01 (0.76, 1.34)

Rate^$^ per 10000 person-year; Crude HR^#^, relative hazard ratio;

† Model was adjusted for age, sex, and comorbidities of hypertension, diabetes, hyperlipidemia, COPD, CAD, stroke, rhinitis, chronic sinusitis, GERD and obesity;

*p<0.05,

**p<0.01
